# Viewing Meaningful Work Through the Lens of Time

**DOI:** 10.3389/fpsyg.2020.585274

**Published:** 2020-10-02

**Authors:** Francesco Tommasi, Andrea Ceschi, Riccardo Sartori

**Affiliations:** Human Sciences Department, University of Verona, Verona, Italy

**Keywords:** meaningful work, meaningfulness, time-based definition, temporal framework, work and organizational psychology

## Abstract

Authors have paid considerable attention to how to define the meaningful work construct. This has led to providing comprehensive definitions in the light of different theoretical frameworks that reflect a degree of contestation within the field. Several of them have proposed definitions linked to the individuals’ pervasive sense of the value of their work. Others have offered descriptions centered on their temporal, episodic nature and emphasizing the individual’s occasional work experience. These definitions reflected a potential temporal condition as well as the variety of time perspectives underpinning the authors’ conceptualizations of the construct. This paper conducted a broad literature review to analyze works that have adopted a temporal framework or supported a time-based definition of the construct. The analysis indicates two different conceptualizations of the construct: as a permanent/steady mindset and as a changeable/episodic experience. As a reflective paper, the present contribution develops an overall framework for views and theories on meaningful work. It reports a critical review on the matter to elevate understanding of meaningful work for further research and applied implications in work and organizational studies.

*“It is sadly true that many jobs are not lovable […] We can and must fight to see that the fruit of labor remains in the hands of those who work, and that work does not turn into punishment; but love or, conversely, hatred of work is an inner, original heritage, which depends greatly on the story of the individual and less than is believed on the productive structures within which the work is done.”* –Levi (1978).

## Introduction

The current turbulent times for the global economy have witnessed increased interest among scholars and authors in the construct *meaningful work* and linked factors. In the wake of the fourth industrial revolution, the pressure on the working status and the constant transformation of labor (Eurofound, 2014) bring the prospect of uncertain and negative consequences for workers as well as for organizations and systems (Schnell et al., 2013). As the most recent research suggests, meaningful work represents a moral and pragmatic concern for all those—individuals, organizations, and systems—who hope to prosper within this plethora of changes and renewed works (Yeoman et al., 2019).

In the field of work and organizational studies, authors aiming to develop theory and to offer practically applicable interventions have tried to find a link between people’s meaningful work and their working and financial conditions. The existing literature, however, renders these aims extremely difficult to achieve. Range of different essential insights have been proposed, suggesting that meaningful work is affected by a multiplicity of factors and conditions, one of which is temporal agency (Bailey and Madden, 2017). We must, therefore, regard meaningful work as a complex phenomenon (Rosso et al., 2010; Dik et al., 2013; Bailey et al., 2019). Furthermore, there is still little agreement on the definition and operationalization of the construct among the scientific communities, and no agreed underlying framework for the development of descriptions of its dimensions (Rosso et al., 2010; Both-Nwabuwe et al., 2017; Bailey et al., 2018).

In their introduction to the special issue of the Journal of Management Studies on meaningful work, Bailey et al. (2019) evoked the *theory of paradox* to report a possible dual nature of meaningful work linked to spatial and temporal agents. According to these authors, the meaningful work construct refers to a pervasive sense of the value of one’s work (Rosso et al., 2010; Tablan, 2019); however, “it may be temporary, partial or episodic” (Bailey et al., 2019). In this vein, there are some examples of definitions of meaningful work characterized by underlying time perspectives. Some authors have insisted on the episodic nature of meaningful work, for example, suggesting that it occurs when “work events, work encounters, or work contexts gain significance, or spiritual value that transform the meaning of work itself” (Madden and Bailey, 2019, p. 152). It is the case of contributions on meaningful work and self-transcendental experience. The self-transcendence concept suggests that an irregular and unusual experience of human potential exists, related to the episodic experience at work of spiritual and social connections between the individual’s inner and the outer lives (Bailey and Madden, 2017). Likewise, there are authors that insisted on the definition of meaningful work as a state of flux and linked to specific events and conditions of work (Mitra and Buzzanell, 2017). Other authors have defined meaningful work in terms of a permanent, or steady, mindset construct, or as the result of the match between a person and specific contents of work and context (May et al., 2004; Rosso et al., 2010; Allan et al., 2019). In this term, authors considered meaningful work as the personal significance when a job provides a sense of self-actualization, self-development, self-connection, and social identity (Pratt and Ashforth, 2003; Rosso et al., 2010; Michaelson et al., 2014).

Although these contributions suggest that there are underlying time-related issues that need to be incorporated in definitions of meaningful work, many questions remain unanswered on the role of time and temporal agency in meaningful work. For example, how can time be included in the definition of the construct? What is the current position of time in the theory of, and empirical research on, meaningful work? To avoid ambiguities over the meaningful work definitions and the various use of time perspectives, this paper intends to organize the literature by means of classifications of studies and seminal review papers deriving from the conventions of the social and human sciences (Lee, 2015; Lepisto and Pratt, 2017). Following others (Sartori et al., 2018), the present contribution aims to conduct a critical review of the literature to elevate the understanding on meaningful work by the definition of a novel framework and proposing a preliminary model of factors subsumed by the construct toward a time-based approach.

### Aims of the Contribution

As noted, authors differentiated aspects of meaningful work into changeable/episodic experience and permanent/steady mindset; thus, respectively, one is considered as a more transient experience to a situation, and the other as a more stable worker’s attribute in experiencing their work. By explicitly approaching meaningful work through the lens of time, the present contribution aims at discussing the nature of this construct.

This is to say that time has been a neglected topic in the study of work, although it is a promising lens for discussing and comprehending work phenomena. In fact, temporal lens and time-based analysis offer an essential framework for “explaining and understanding organizational behaviors (constructs)” and “it focuses our attention on new classes of independent and dependent variables” (Ancona et al., 2001, p. 646). Other, similar, contributions suggest that this unique framework can “sharpen the lens” for theory and research building within work and organizational research (Bakker, 2010; Sonnentag, 2012; Navarro et al., 2015; Cole et al., 2016; Eldor et al., 2017; Pinto, 2017). Indeed, this view seems to enable us not only to avoid uncertainty around the conceptualizations of work phenomena but also (a) to revise a number of perspectives, (b) to place them in a common framework, and (c) to understand the objects of study as well as the relations between the variables. For example, classes of variables would be categorized differently in the wake of their modification and trajectories over time, hence revealing opportunities and new directions for research. It affects not only the definition, classification, and operationalization of variables but also our thinking about understanding psychological and working phenomena (Ancona et al., 2001; Roe, 2008).

In the case of meaningful work, it can be noted that this approach can help to understand the situational conditions (i.e., changeable and stable) of meaning in work (Tummers and Dulk, 2011; Tummers and Knies, 2013; Bailey and Madden, 2017). Moreover, it can serve as a framework to comprehend how psychological, working, and environmental factors interact, both *per se* and with regard to the experience and presence of meaning (Xanthopoulou et al., 2009; Bakker, 2014; Yeoman, 2014a; Bailey et al., 2017a; Fletcher et al., 2018). Accordingly, the critical review intends to discuss in depth why, when, and how meaningful work is defined and in particular what defines it as a personal characteristic of an individual’s sense of value. This can be, for example, in one’s own narration of one’s self at work (Manuti et al., 2016) or a general characteristic of the individual, similar to a personal trait (Wrzesniewski et al., 1997; Wrzesniewski, 2003; Lysova et al., 2019). Likewise, why, when, and how meaningful work is defined and what defines it in terms of the personal and episodic *state of meaning* relate to the intra-individual fluctuations associated with daily experiences at work (Muzzetto, 2006; Thompson and Bunderson, 2007; Ruswahida, 2014).

Given these possibilities, this article addresses the research questions on meaningful work taking into account a time-based approach. After presenting a broad body of literature, the two distinct natures of meaningful work construct are presented, i.e., steady mindset and episodic, by outlining the existing classifications and discussions on meaningful work research within the social sciences. As follows, the contribution discusses the dual nature of meaningful work providing a critical review of factors that influence meaningful work toward the lens of time. Implications for research and practice are latter presented.

## Meaningful Work and Time

### Definitions of Meaningful Work

In the literature, there is no broad consensus about the definition of meaningful work, so, in order to obtain a comprehensive view of the role of time, and to conduct further exploration of the separate topics and subtopics, it is helpful at the outset to establish an overview of how authors discussed the construct. In this, two main objects of analysis are relevant: the discrimination of the terms used and the array of perspectives on how to define and measure meaningful work (Rosso et al., 2010; Lepisto and Pratt, 2017).

#### “Meaning of” and “Meaningfulness”

Rosso et al. (2010) noted that meaningful work has been defined and operationalized in various ways and using interchangeable terms (Rosso et al., 2010; Allan et al., 2019). Therefore, the authors distinguish accurately between the following terms: *meaning of*, *meaningful, meaningfulness*, and *meaning in/at*. The term “*of*” generally refers to what something signifies to one individual. Hence, using this terminology indicates the cognitive process by which an individual interprets and attaches a meaning to their work (Wrzesniewski, 2003; Willner et al., 2019), although it can have a different value (Lepisto and Pratt, 2017) pertaining to when work *per se* is at issue (Schnell et al., 2013). Meaningful work, meaningfulness, and meaning in/at refer to significance, subjective experience, and perception of the value of work (Lips-Wiersma and Morris, 2009; Rosso et al., 2010; Schnell et al., 2013; Allan et al., 2019).

#### Conceptualizations of Meaningful Work

The recent work of Bailey et al. (2018) suggests a substantial way for classifying the conceptualizations of meaningful work literature. These authors have proposed a review of the existing empirical evidence on meaningful work, in which they discussed an original viewpoint on the boundaries of current knowledge. They scrutinized the perspectives of 71 articles and argued that the underlying theoretical framework of the collected empirical studies generally referred to positive psychology (i.e., Oldham and Hackman, 1981) and the literature on spirituality and “calling.” As they indicated, some authors proposed definitions within the job characteristic model and conceptualized meaningful work as a core psychological state of work motivation. Others looked at studies that examined models around “workplace spirituality” in which the emphasis is on the role of organizations to enable human flourishing by sustaining people’s need for an inner life (Milliman et al., 2017; Bailey et al., 2018). Bailey et al. (2018) grouped all the approaches to meaningful work in a third strand of research, the humanistic perspective, to classify those contributions that principally define meaningful work as inherently subjective. In this class, some authors discuss meaningful work as the effect of the human ontological will for meaning (e.g., in reference to the classical works in the humanistic perspective, as Jung, 1933; Frankl, 1985). Others define it as a eudemonic psychological state as the result of the individual’s broad judgment on their life and work.

Bailey et al. (2018) proposed a useful framework for classification of the numerous definitions of meaningful work and offered a comprehensive view of the current research strands; however, how a time-based approach could be included in these classifications remains uncertain. Moreover, in the literature, there are other seminal works, in which overreaching viewpoints and theories are proposed. Although they offer an essential view to comprehend the literature on meaningful work, they do not support the treatment of the research in terms of time-based definition.

### Meaningful Work Through the Lens of Time

A broad exploration of the literature has been made referring to the time-based approach. According to the aim of the study, this review explored meaningful work through the lens of time by incorporating different sources (e.g., research papers, book chapters) and various research fields (e.g., psychology, sociology, organizational studies). Thus, time is present in separate meanings within the contributions on meaningful work collected (see Table 1). It emerged as an underlying factor in the definition of the construct, both in everyday work and in atypical work contexts as well as in precarious employment and long-term jobs. In fact, time and temporality are discussed concerning jobs inherently meaningful and not and there is an ambiguous condition that concerns whether meaningful work consists in episodic experiences or in a pervasive sense of the value of one’s work, i.e., whether it occurs in the course of time, or whether a degree of stability is present or absent (Bailey et al., 2017b; Lavy and Bocker, 2018; Bailey et al., 2019). For example, some authors examine the episodic occurrences of meaningful work in relation to specific contexts and conditions (e.g., liminal experiences, Toraldo et al., 2019). Among them, such authors present the episodic nature as flux experiences (Mitra and Buzzanell, 2017) or by reference to the working and psychological conditions at work, which predict the occasional experience (Scott, 2019). Others explicitly report meaningful work as a stable characteristic of the subject, as a specific subjective concern of individuals, which is different from the experience of meaningful work experiences (e.g., psychological perception vs. significance, Lavy and Bocker, 2018; global meaning vs. situational, Park and Folkman, 1997).

**TABLE 1:** Meaningful work through a time-based lens.

Time-based interpretations	Authors	Definition
Steady mindset	Pratt and Ashforth (2003, p. 311)	“[…] work and/or its context are perceived by its practitioners to be, at minimum, purposeful and significant. […] This perception may derive from the intrinsic qualities of the work itself, the goals, values, and beliefs that the work is thought to serve, or the organizational community within the work is embedded”
	Barrett and Dailey (2018, p. 284)	“[…] constructions of meaningful work are constituted in emergent moments of interaction, produced by historical acts, and derived from a wide array of cultural discourses (Kuhn et al., 2008; Wieland, 2011).”
	Chalofsky and Krishna (2009, p. 197)	“Meaningful work is not just about the meaning of the paid work we perform; it is about the way we live our lives. It is the alignment of purpose, values, and the relationships and activities we pursue in life”
	Allan et al. (2019, p. 16)	“Without stable job characteristics, people’s sense of meaningful work may be the thread that runs between temporary positions”
	Lips-Wiersma and Morris (2009, p. 505)	“[…] meaningful living requires paying attention to both “doing and being” and both “self and other””
	Cheney et al. (2008, p. 144)	“meaningful work may be conceptualized as a job, a coherent set of tasks, or any endeavor requiring mental and/or physical exertion that an individual interprets as having a purpose (see also Pratt and Ashforth, 2003)”
	Michaelson et al. (2014, p. 79)	“[.] how an individual view him or herself (i.e., her or his identity) strongly influences how she or he views his or her work. Alternatively, the more task-centered and more objective focus on meaningfulness explores job characteristics in work that are perceived to be meaningful or that support the individual pursuit of meaningfulness at work”
	Mainemelis (2002, p. 235)	“[.] timelessness is facilitated, among other factors, by intrinsic motivation, autonomy, and meaningful work, and is hindered by extreme pressures and distractions in the work environment”
Episodic	Bailey and Madden (2017, p. 2)	“meaningfulness arose episodically through work experiences that were shared, autonomous and temporally complex. Schutz’s notion of the “vivid present” emerged as relevant to understanding how work is rendered meaningful within an individual’s personal and social system of relevance”
	De Boeck et al. (2019, p. 530)	“untapped potential as a subjective temporal experience that can make work more, or less, meaningful from the perspective of the individual employee by functioning as a cognitive bridge between the present and the future”
	Fletcher and Schofield (2019, p. 23)	“the way in which meaningfulness ‘emerges from an appreciative or reflective act in which the significance of the moment is perceived within a wider timescape”
	Matz-Costa et al. (2019, p. 1127)	“Exploring such within-person changes enables an examination of proximal (i.e., state-like as opposed to trait-like) predictors of perceived meaningfulness, such as person-specific states or situational features that are present at a certain point in the day. Such research is needed to investigate the full phenomenological experience of work meaning and to clarify the underlying dynamics of deriving meaning from one’s work”
	Mitra and Buzzanell (2017, p. 70)	“meaning-making of work [is] constantly in flux, rather than a static frame, shaped by the constraints facing them”
	Scott (2019, p. 17)	“participants […] reported a sense of meaningfulness about their work, and stories about mastery, having an impact on others, reaching potential – stories of agency – characterized their responses”
	Madden and Bailey (2019, p. 155)	Further empirical research supports this temporal aspect of meaningfulness, to show that it is not a steady or sustained experience but is experienced “in transcendent moments in time”
	May et al. (2019, p. 364)	“Experiencing meaning is inherently less than stable or constant and can be seen to involve natural tensions”
	Toraldo et al. (2019, p. 648)	“new work forms invoke meaningfulness beyond traditional economic incentives while not excluding instrumental motives. [.] by linking voluntarism with the temporary nature of festivals, we contribute to understanding how such events shape meaningfulness [.] acknowledging the micro-emancipatory moments”
Steady mindset vs. Episodic	Lavy and Bocker (2018, p. 1494)	“the sense of meaning at work is not a completely stable, permanent condition, but rather a frequent occurrence, which can be renewed daily (Pratt and Ashforth, 2003), and may, therefore, be affected by events and experiences at work (Clausen and Borg, 2011)”
	Bailey et al. (2019, p. 495)	“meaningfulness is a pervasive sense of the value of one’s work, yet it is also linked with spatial, temporal and material contexts which may be temporary, partial or episodic”
	Bailey et al. (2017b, p. 427)	“whether meaningfulness is momentary and similar in functioning to such experiences as flow (Csikszentmihalyi, 1990), linked with longer-term fluctuations depending on work conditions, akin to engagement (Kahn, 1990), or whether it is a relatively stable, subjective state”
	Park and Folkman (1997, p. 116)	“Global meaning encompasses a person’s enduring beliefs and valued goals. […] meaning as “the cognizance of order, coherence, and purpose in one’s existence, the pursuit and attainment of worthwhile goals, and an accompanying sense of fulfillment” […] situational meaning as the meaning that is formed in the interaction between a person’s global meaning and the circumstances of a person-environment transaction”

#### The Dual Nature of Meaningful Work

By the interpretation of definitions of meaningful work through the lens of time and a time-based synthesis approach, two main categories of meaningful work emerge, namely, as a stable subjective mindset of a worker and as an experience that can occur in specific psychological and working conditions. These categories related both to the subjective experiences of time and the objective nature and facets of time (e.g., the passage of clock time or the time needed for particular tasks). Meaningful work as a stable/permanent mindset or as changeable/episodic experience appear in the structuration of the continuous axis of time, on which events and conditions are arranged—following the proposition of *real-time* in the Aristotelian view as a “physical and quantifiable entity” (Aristotele. 4AD, 1991).

On the one hand, the internal significance of meaningful work would shape the quality of time and work experience. As such, meaningful work as a steady mindset refers to the worker general significance attached to a job that is meaningful *per se*, e.g., when a job is a source of meaningfulness, as a pervasive sense of the value of one’s work (Mainemelis, 2002; Cheney et al., 2008; Michaelson et al., 2014; Barrett and Dailey, 2018). For example, Allan et al. (2019) suggested that “without stable job characteristics, people’s sense of meaningful work may be the thread that runs between temporary positions” p. 16. This general significance attached to work itself would be gained by the retrospective and cognitive judgments of the inner individual experience and knowledge (Kahneman et al., 2006). The resulting global meaning in work would be a factor in the stable characteristics of individuals that affect both the individual’s work behavior and perceptions of work experiences and aspects of the job and its organization (Park and Folkman, 1997; Mainemelis, 2002; Allan et al., 2019). In line with this thesis, meaningful work is discussed to be as a steady mindset by other authors, e.g., Bailey et al. (2017b), who show how the presence of a global judgment of meaningful work would be predictive of psychological states at work (e.g., job satisfaction, Barrett and Dailey, 2018). These authors agree with the theoretical framework discussed by Rosso et al. (2010), comprising significance, beliefs, definitions, and value attached to work by individuals—where work is a significant component of human activity and lives (Pratt and Ashforth, 2003; Lips-Wiersma and Morris, 2009; Lavy and Bocker, 2018).

On the other hand, experiences of meaningful work consist in episodic experiences as referred to the individual’s daily work experiences in which different events and conditions take place. For example, following the definition of time by Aristotle, events occur along an axis by which individuals allocate their (working and) psychological conditions that influence their meaning (in/at work) experience (Bailey and Madden, 2017; Lavy and Bocker, 2018; Matz-Costa et al., 2019). Authors who discuss the state and episodic nature of work argue that meaningful work could be experienced as a temporary embedded subjective experience where past, present, and future coexist. This can occur in a sort state of a constant flux (Mitra and Buzzanell, 2017), between time and space, outside the common working norms (Toraldo et al., 2019), or it can be linked to specific, isolatable working and psychological conditions (Bailey and Madden, 2017; Lavy and Bocker, 2018; Fletcher and Schofield, 2019; Matz-Costa et al., 2019; Scott, 2019; Yeoman et al., 2019). Moreover, such authors define meaningful work as episodic experience as if it occurs in the course of time or it unfolds over time. In fact, meaningful work has been considered as the end of the meaning-making process by which meaningfulness can unfold through the real physical and quantifiable time. In this vein, the tensions occurring over time between one individual and his/her job, organization, and socio-political context can result in different states, such as meaningful work. Therefore, there can be fluctuations of the degree of meaningful work experience as well as variations of the presence/absence of meaning in reference to the past, present, or to the being stuck in an eternal present (De Boeck et al., 2019) or pointless conditions (Yeoman et al., 2019).

In general, the construct of meaningful work has been characterized by using a variety of time perspectives ranging from the steady mindset/permanent conceptualizations to episodic/occasional definitions. As seen, time represents the continuous axis on which the phenomena of life and work appear within different contexts and situations. Onto this objective, physical and measurable agency individuals attach subjective meaning and have personal experiences. Therefore, meaningful work may be shortly defined, and considered, as a positive “subjective experience of existential significance” (Both-Nwabuwe et al., 2017, p. 7) that results in, or is fostered and maintained by, central main pathways comprehending individual, organizational, and socio-political factors (Lepisto and Pratt, 2017). This experience may be a steady mindset when a work is experienced and perceived as meaningful as it responds to the individual’s quests for meaning in their work and life, and it provides a sense of self-actualization, self-development, self-connection, and social identity (Pratt and Ashforth, 2003; Rosso et al., 2010; Michaelson et al., 2014; Lepisto and Pratt, 2017; Martela and Pessi, 2018). Likewise, episodic experience of meaningfulness regards the existential experience that can occur in a specific time “such as person-specific states or situational features that are present at a certain point in the day” (Matz-Costa et al., 2019, p. 70), “which can be renewed daily (Pratt and Ashforth, 2003), and may, therefore, be affected by events and experiences at work” (Lavy and Bocker, 2018, p. 144).

## Toward the Dual Nature of Meaningful Work

**FIGURE 1:**
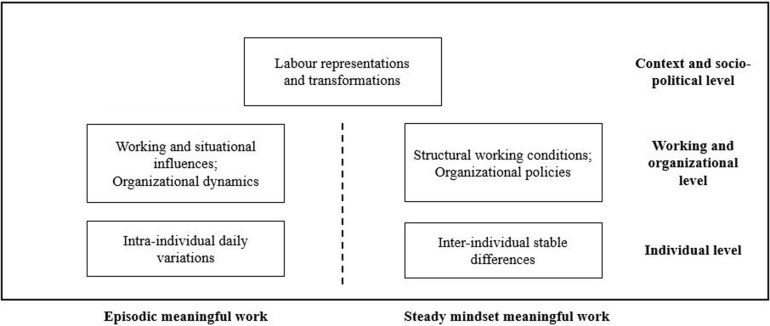
A preliminary model of meaningful work and the three levels of factors toward the lens of time.

In the reviewed literature, authors discussed meaningful work by explicitly referring to identifiable factors that can affect the way work can be meaningful both as a steady mindset or as an episodic experience. These factors appear to be differentiated at three levels, namely, (a) individual level (Wrzesniewski et al., 1997; Allan et al., 2019; Lysova et al., 2019), (b) working and organizational level (Schnell et al., 2013; Bailey et al., 2017b; Mitra and Buzzanell, 2017; Lysova et al., 2019), and (c) cultural and socio-political level (Yeoman, 2014a; Lepisto and Pratt, 2017; Bendassolli and Tateo, 2018; Yeoman et al., 2019). This result pointed out the fact that, although authors have adopted separate time-based definitions of the construct, meaningful work should be considered by looking at the various factors that can contribute to its presence. This evidence initiates a deeper reflection suggesting a possible novel framework of meaningful work toward the lens of time (see Figure 1).

According to the comprehension of the dual nature of meaningful work, the following sections advance the propositions for future explorations of the factors subsumed by meaningful work with a deeper focus on time as a full frame for theory-building. This proposal constitutes a preliminary working model of factors that contribute to the presence of meaningful work. Moreover, the aim is to present a conceptual framework on the dual nature of meaningful work that will help both authors and practitioners in identifying the variety of aspects that this construct subsumes. Thus, the contribution examines meaningful work as permanent/steady mindset and meaningful work as a changeable/episodic experience by looking at the macro-levels of factors identified, succinctly: individual, organizational, and contextual levels. Beside the theoretical implications, this framework supports a different focus on work and workers’ aspects on which practitioners and researchers can focus on.

### Individual Level

Meaningful work can be referred to a transient experience as a positive subjective experience of existential significance that will depend on the daily intra-individual and environmental conditions. Likewise, meaningful work can be a more stable worker’s attribute in experiencing their work where individual differences play an important role in the creation of stable significance attribution. Firstly, meaningful work is, then, conceptualized assuming its episodic nature and linked intra-individual daily variations (Oldham and Hackman, 1981; Tims et al., 2016; Lepisto and Pratt, 2017; Vogel et al., 2019). This concept has been discussed in both qualitative and quantitative studies. For example, the qualitative research by Bailey and Madden (2017) showed how the participants had specific experiences of connection with others and their jobs, reporting episodic experiences of self-transcendental experience suggesting an episodic occasion of meaningful work. In their longitudinal research using diary studies, Matz-Costa et al. (2019) found that the daily perception of meaningful work was related to the emotional states and behavior at work as the job crafting behavior. In particular, the job crafting behavior regards the individual ability to enact organizational behavior by which they can change their thoughts about their job and their working experiences (Tims et al., 2016; Costantini et al., 2017b, 2019; Lavy and Bocker, 2018). Moreover, Allan (2017) found that task significance prompted the experience of meaningful work in a longitudinal setting, which highlights the insights of Kahn (1990), for whom the fluctuations of meaning depended on the perceived work conditions (Fletcher et al., 2018). Similarly, in the recent studies on work engagement (Xanthopoulou et al., 2009; Bakker, 2014; Bailey et al., 2017b; Fletcher et al., 2018), the episodic experience of meaningful work is seen to show daily fluctuations during the working day due to the ambient psychological and working conditions, which makes it a different phenomenon from the steady mindset explored above.

Secondly, from the humanistic perspective (based on the seminal classical works of Frankl, 1985, and Jung, 1933), it is universal in human beings to search for and attribute meaning. The analysis of the subjective meaning of work revealed that it can be evaluated as a steady mindset in terms of both presence and absence and the degree of its stability (Steger et al., 2006; Lips-Wiersma and Morris, 2009; Devivere, 2018; Martela and Pessi, 2018; Allan et al., 2019; Lysova et al., 2019; Yeoman et al., 2019). The level of stability links to a work that is experienced and perceived as meaningful as it responds to the individual’s quests for meaning in their work and life. Therefore, it is linked to the inter-individual stable differences (Rothmann et al., 2019) as the dispositional signature (Lysova et al., 2019), cultural belongingness (Lepisto and Pratt, 2017; Bendassolli and Tateo, 2018), work values (Consiglio et al., 2017), work orientation, and work narratives (Wrzesniewski et al., 1997; Scott, 2019). Generally, authors writing in this area have discussed one individual’s seeking for meaning as positive (Rosso et al., 2010), a eudemonic state (Steger et al., 2012), and an inherently human quest: “a condition of being human to make meaning” (Lips-Wiersma and Morris, 2009). According to the humanistic perspective, the quest for meaning cannot be supplied by organizations or context, although it is ostensibly linked to the socio-political context (Tummers and Dulk, 2011; Yeoman, 2014b). In this vein, meaningful work can vary between each person as well as be permanent along the axis of objective time, in a way that lasts for a long time.

### Working and Organizational Level

From the point of view of the working and organizational features, meaningful work is still discussed in terms of its dual nature, stable and episodic. At the individual level, steady meaningful work is linked to the organization’s sources of meaning and to the particular features of the job. Type, quality, and amount of work are relatively stable characteristics of a job and organizations, namely, working structural conditions (Xanthopoulou et al., 2009; Bakker, 2010, 2014). The meanings that a person attached to their job links to their internal dispositions, and the characteristics of an organization, as organizational policies, can prompt a sense of value at work, e.g., *belonging*, *significance*, *coherence*, and *direction*, which are core components of meaningful work (Rosso et al., 2010; Schnell et al., 2013). Moreover, on a daily basis, the features of the job can vary and show different sources of meanings, depending on both the working and situational influences, e.g., daily demands and resources (Martela and Riekki, 2018), and organizational dynamics. The sense of autonomy and relatedness, for instance, can be different from 1 day to another and from one task to another, Similarly, the significance of the tasks at work (Allan, 2017) can prompt differences in the experience of meaningfulness, i.e., episodic (Wellman and Spreitzer, 2011). In this case, working and situational variations and organizational dynamics may foster or inhibit daily significant experiences.

At the organizational level, the sources of meaning relate to the stable characteristics of the organization’s culture, policies, and practices. The style of leadership can shape the emotional atmosphere and hence the experience of positive emotion and meaningful work (Tummers and Knies, 2013; Carton, 2018). Workplace spirituality and organizational democracy can foster a sense of belonging and can shape meaningful work experience (Yeoman, 2014b; Schnell et al., 2019; Weber et al., 2019), but episodic meaningfulness and meaninglessness can also be associated with the low-quality leader–member exchange relationships (Tummers and Knies, 2013; Bailey et al., 2017b; Bendassolli, 2017a), which can prompt a sense of inter-individual solidarity and, consequently, the sense of meaning. State affects, affective events, and discrete emotions in the workplace, as reported in the study of Matz-Costa et al. (2019), can also determine fluctuations in the meaningfulness of work. Emotions in the workplace have received much attention in the field of organizational psychology and organizational behavior (Ashkanasy et al., 2002). Recent works have reported evidence of the links between the personal, interpersonal, and organizational levels (Ashkanasy et al., 2002; Ashkanasy and Humphrey, 2011). Since the multi-level model of emotion in organizations explains how different organizational dynamics have their effect on the worker, at all levels from the within-person variations (i.e., affective events) up to broad environmental changes (i.e., the emotional climate), variations of meaningful work as a mediator of positive behavioral outcomes can be measured and observed (Matz-Costa et al., 2019).

### Context and Socio-Political Level

In the literature, several authors discussed conditions of and transformation of work—all of which were difficult to assess—context and socio-political influences as important categories in studying meaningful work. The socio-political context includes various factors such as the access to decent work (Duffy et al., 2017), culture (Bendassolli and Tateo, 2018), and political reforms, and labor transformations and representations (Schwartz, 1982; Gill, 1999; Mitra and Buzzanell, 2017; Barrett and Dailey, 2018; Yeoman et al., 2019; Tommasi et al., 2020). The combination of these factors shapes the way individuals attach meaning to their work. In the current context of temporary and difficult jobs and socio-political changes, some authors hypothesized that individuals can find a meaning crafting their experience to gain an experience of meaningful work (Wrzesniewski and Dutton, 2001; Rosso et al., 2010; Berg et al., 2013; Mitra and Buzzanell, 2017). Existing literature reports how the economy and society structure jobs and organizations in a top-down manner, with a focus on the stable characteristics of labor conditions that highlight the need for future research on the experience of meaningful work within a more substantial temporal lens (MOW International Research Team, 1987; Willner et al., 2019). As noted by Thompson (2019), the literature in the field mostly overlooks the relevance of macro-aspects of the institutions on shaping the opportunities for meaningful work. While pointing out the consequences of a meaningful work (e.g., spillover effects on civic participation), he argues that three paths of arrangements in terms of labor representations and labor transformations can be taken for promoting meaningful work at the institutional level. These are: (a) encouraging social actors to cooperate with the state in creating meaningful work; (b) renewing the balance of power, straightening the role for labor representations; and (c) beginning to reframe the social discourse on meaningful work. Although Thompson remarks the complexity of studying *work* and *organization* (Friedman, 1946/1955), empirical findings have shown how individuals regularly deal with socio-political conditions, i.e., labor representations and transformations, during the meaning-making process (Mitra and Buzzanell, 2017) and enact behavior (i.e., job crafting) that changes their work conditions, mindset, and organizational behaviors (Wrzesniewski and Dutton, 2001; Spencer, 2015; Ward and King, 2017).

Mitra and Buzzanell (2017) support the use of the “continuous axis of time” when discussing political implications for meaningful work. They regard as socio-political context those pressures that foster the internalization of preferred self by workers who negotiate their control on the meaning-making process. Since these factors occur in a temporal tension—during the meaning-making process—meaningful (as meaningless) work reflects its temporal nature. Meaningfulness and meaninglessness unfold in time, time that is closely related to the (complementary) objective time in which workers make their work and life experiences. This suggests two strands of research. Firstly, authors could seek to understand how meaningful work historically changes in the light of the socio-political changes that take place among the factors that contribute to the account-making of work (Shantz et al., 2015; Allan et al., 2017; Bendassolli, 2017b; Lepisto and Pratt, 2017). Secondly, in the current economic times, authors can consider different kinds of work (e.g., precarious employments, Patulny et al., 2020) to explore further the assessment of account-making the presence of the four significant sources of meaning in work (Twenge et al., 2010; Yeoman et al., 2019).

The authors who suggested a temporal lens referring to the socio-political level have also explored organizational behavior in conditions of (not) decent work (Duffy et al., 2006; Di Fabio and Kenny, 2016). Future research may examine how individuals deal with temporary jobs, precarious employments, and uncertain working conditions due to the economic changes, and how individuals enact behavioral changes in order to experience meaningful work (Wrzesniewski and Dutton, 2001; Berg et al., 2013; Demerouti and Bakker, 2014; Allan et al., 2020; Patulny et al., 2020). Indeed, examining these issues would enlarge our knowledge of the dual nature of meaningful work, establishing evidence that the construct can be conceptualized as inherently distinct from other psychological dimensions (Chalofsky and Krishna, 2009; Berkman et al., 2017).

## Further Considerations

Fundamental questions about *time* have been part of a long story in philosophy and more widely in the human sciences. Only a few authors—in and out of the field of meaningful work—have included time in theoretical or empirical studies. Time is now, however, receiving more attention within psychology and the social sciences (Roe, 2008; Sonnentag, 2012; Navarro et al., 2015; Cole et al., 2016; Pinto, 2017; Tommasi, 2020). Researchers are arguing for the use of time in theory and practice, seeking resolutions to the disagreements about the phenomena of work (Ancona et al., 2001; Cunliffe et al., 2004). Indeed, time and the order of time are significant concerns within the study of people’s lives and their work (Eldor et al., 2017).

In 1911, Taylor published his book on the organization of working hours and workers, *The Principles of the Scientific Management*, in which he proposes a view of time as objective and measurable and where he discusses the industrial process as an “hegemonic discourse centering on precision, control, and discipline” (Taylor, 1911/1970; Hassard, 2000, cited in Bailey and Madden, 2017, p. 4). Indeed, the industrialization process “arose out of the measurement of work. It’s when work can be measured, when you can hitch a man to the job, when you can put a harness on him, and measure his output in terms of a single piece and pay him by the piece or by the hour, that you have got modern industrialization” (Bell in Marcuse, 1964/1991, p. 32). In this vein, following the Aristotelian argument, time is seen as essentially objective, *physical* and *quantifiable* (Rämö, 2004). Individuals make actions on a continuous, linear, physical axis that is independent of humans. This is distinct from the subjective view of time, in which the themes of *past, present*, and *future* are seen in the experience and meanings of individuals (Hassard, 2001; Eldor et al., 2017). Although this common distinction is part of extensive discussions within different disciplines, we can say that subjective and objective time can be seen as complementary (Ancona et al., 2001). Subjective time inevitably relates to the perception of objective time. However, some aspects of the subjective experience of time (e.g., the passage of the clock time, working hours, etc.) could give time different meanings and perceptions (Eldor et al., 2017). For example, during working hours, the speed of time may depend on whether experience at work is seen as meaningful (Bailey and Madden, 2017) or not (Hassard, 2001; Cunliffe et al., 2004; Eldor et al., 2017).

The present paper aimed to propose a critical perspective on meaningful work through a time-based definition approach. Although the existing literature has made significant steps in the field, the neglected role of time in the conceptualization of meaningful work represents a challenge for the current research. This paper has tried to respond to the call for a wider model of the construct, building on the need to conceptualize meaningful work according to the time view (Bailey et al., 2019). Moreover, since the model of a dual nature of meaningful work reveals a different focus on work and workers aspects based on the different levels on which focus on, research and applied implications must be discussed.

### Implications of the Contribution

Considering that most of the people have to spend at least 40 h per week, for 40+ weeks per year, for 40+ years of their life, at work, the presence of meaningful work becomes fundamentally essential for workers, organizations, and systems. Likewise, it is relevant for researchers and practitioners to understand how and to what extent the temporal conditions of the construct occur in order to propose applied interventions for individuals and organizations.

Most people search for meaning in a job (Frankl, 1985; Devivere, 2018), for something more than a job “where you go home and maybe go by a year later and you don’t know what you’ve done” (Terkel, 1972, p. 32). The attribution of meaning, its quality and contents, is mainly subjective, as is one’s orientation to one’s work (Wrzesniewski et al., 1997; Wrzesniewski, 2003; Lepisto and Pratt, 2017), but sources of meaningful work are reliably correlated with the workplace and the working activities (Michaelson et al., 2014; Weber et al., 2019; Yeoman et al., 2019). Viewing meaningful work through the lens of time leads to consider its dual nature. The broad literature review has considered conceptualization underlying a temporal framework or supporting a time-based definition of the construct. The analysis indicated two different conceptualizations of the construct: as a permanent/steady mindset and as a changeable/episodic experience. As discussed above, the characteristics of meaningful work can be either stable or changeable and subsume the presence of three classes of factors that contribute to its presence. In this vein, a preliminary model of the dual nature of meaningful work and related factors has been proposed with the intention to support further exploration of these initial prepositions.

#### Applied Implications: Meaningful Work Interventions

These conclusion can yield possible interventions for workers and organizations. Indeed, taking stock of time in the definition of meaningfulness and establishing evidence of stable and episodic experiences suggests possible applied implications.

How to understand the possible twists and turns of training interventions is a crucial question for practitioners attempting to improve organizational conditions (e.g., workers’ well-being or motivations and personal improvement, Ceschi et al., 2017; Sartori and Tacconi, 2017). Through the lens of time, environmental and individual variables show a more profound complexity (Navarro et al., 2015; Tommasi, 2020). Using the distinction advanced here, within the frame of the three groups of factors suggested, would offer an essential contribution in devising applied research programs and training interventions. Indeed, the studies analyzed suggest that the ways in which meaningfulness can arise depend on several factors (Chalofsky and Krishna, 2009; Lee, 2015; Costantini et al., 2017a; Bailey et al., 2018). By adopting the framework of the three levels of analysis (i.e., individual, organizational, and contextual), practitioners can deal with any possible discrepancies between interventions’ intentions and workforce expectations by approaching the phenomenon more innovatively, in particular by specifying both the intervention targets and the classes of agents to be addressed.

Firstly, focusing on the permanent aspects of meaningful work will lead practitioners to consider interventions intended to align workers’ expectations with the environmental context at the individual level. For example, discussions on *existential indifference* as presented by Schnell (2010) in the study of meaning in life, showed that not all individuals are interested in the attribution of meaning to their lives. If considered in the workplace, the presence of existential indifference within workers can reflect a discrepancy at work when planning meaningful work interventions. Indeed, the details of the intervention should be planned by reference to the individual’s characteristics, assessed in pre-training conditions. This discrepancy may show the challenges of meaningful work intervention in which workers have no interests in receiving a training intervention. Nowadays, the literature on how workers respond to meaningful interventions is generally silent (Fletcher and Schofield, 2019). Therefore, a pre-intervention analysis of the participants’ needs is helpful to tailor training.

Secondly, the focus on the job and the organization suggests that, to be appropriate and meaningful, interventions should consider those working and organizational factors that are permanent and not-easily changeable. The *rhetoric* of meaningful work intervention may be misunderstood by workers when job quality and organizational conditions cannot be addressed. Ideally, training intervention should focus on this distinction between the more stable working conditions and the changeable. For instance, the quality of a job seen through a temporal lens is changeable in the medium or long term (Roe, 2008). Job quality is a more stable aspect of one individual’s context than team climate and leadership, so programs to create specific interventions intended to foster meaningful work will be more effective if they include attention to the stable and changeable characteristics of both job and organization.

Thirdly, practitioners devising interventions should also consider the broader societal context and how individuals reflect and process meanings in their working conditions. Socio-political factors play a crucial role in shaping meaningful work. Poor work conditions (e.g., precarious jobs) and complex societal dynamics (e.g., labor transformations) are of course difficult to address. For example, Fletcher and Schofield (2019) have detailed the effects of interventions for meaningful work, analyzing and reporting the influence of the broader socio-political context and working environment. They discussed how the results of Brexit during the period of training had significantly and negatively impacted on participants. On the basis of their findings, they advocate for a broader-based reflection on meaningful work interventions, linking them with all aspects of the context of the work: individual, organizational and socio-political context. In those programs that do not take this on board, there is the risk of abusing the rhetoric of meaningful work, avoiding the reality of the working environment and, consequently, running ineffective intervention programs.

According to the dual nature concept of meaningful work and the proposed model of factors subsumed, it can be suggested that researchers and practitioners should adopt a wide-open lens for tailoring training (Eodice et al., 2019) that takes full account of the views of the individuals involved and of the relevant organizational and contextual factors (Bailey et al., 2018; Fletcher and Schofield, 2019; Yeoman et al., 2019).

## Conclusion

It is apparent that the proliferation of technology changes and globalization coupled with labor market deregulation, precarious employment, and profit maximization will increase in the future, affecting workers, organizations, and systems. Thus, the constant labor and economic transformation call scholars and authors for putting effort in sustaining the quest for meaningful work. As with all the literature in the field, the present contribution hopes that the proposed preliminary model would help researchers and practitioners to improve job quality and support individual lives and well-being. Although the contribution is no more than a critical calling for several studies to examine these ideas in more theoretical and empirical detail, it does have some inevitable limitations. The focus on a temporal framework reflects a limitation in itself because there are undoubtedly several relevant classes of agents in the spatial context. Therefore, future research synthesis might examine together both the temporal lens and spatial agents, examining the interactions between the two.

## Author Contributions

FT and RS developed the concept behind the manuscript and conducted the review. RS and AC contributed with their previous works and experiences in the discussion. FT wrote the manuscript and the co-authors edited the final version. All authors listed have made a substantial, direct and intellectual contribution to the work, and approved it for publication.

## Conflict of Interest

The authors declare that the research was conducted in the absence of any commercial or financial relationships that could be construed as a potential conflict of interest.
