# Corrigendum: Viewing Meaningful Work Through the Lens of Time

**DOI:** 10.3389/fpsyg.2020.616916

**Published:** 2020-11-16

**Authors:** Francesco Tommasi, Andrea Ceschi, Riccardo Sartori

**Affiliations:** Human Sciences Department, University of Verona, Verona, Italy

**Keywords:** meaningful work, meaningfulness, time-based definition, temporal framework, work and organizational psychology

In the original article, the reference for ^**^(Costantini et al., 2017b)^**^ was incorrectly written as ^**^Costantini, A., Riccardo, S., and Andrea, C. (2017b). “Framing workplace innovation through an organisational psychology perspective: a review of current WPI studies,” in *Aligning perspectives on health, safety and well-being. Workplace innovation: Theory, research and practice*, eds P. R. A. Oeij, D. Rus, and F. D. Pot (Cham: Springer), 131–147. doi: 10.1007/978-3-319-56333-6_9^**^. It should be ^**^Costantini, A., Sartori, R., and Ceschi, A. (2017b). “Framing workplace innovation through an organisational psychology perspective: a review of current WPI studies,” in *Aligning Perspectives on Health, Safety and Well-being. Workplace Innovation: Theory, Research and Practice*, eds P. R. A. Oeij, D. Rus, and F. D. Pot (Cham: Springer), 131–147. doi: 10.1007/978-3-319-56333-6_9^**^.

In the original article, the reference for ^**^(Costantini et al., 2019)^**^ was incorrectly written as ^**^Costantini, A., Andrea, C., and Riccardo, S. (2019). “The theory of planned behaviour as a frame for job crafting: explaining and enhancing proactive adjustment at work,” in *Theoretical Approaches to Multi-Cultural Positive Psychological Interventions*, eds L. Van Zyl, and S. Rothmann, Sr. (Cham: Springer), 161–177. doi: 10.1007/978-3-030-20583-6_7^**^. It should be ^**^Costantini, A., Ceschi, A., and Sartori, R. (2019). “The theory of planned behaviour as a frame for job crafting: explaining and enhancing proactive adjustment at work,” in *Theoretical Approaches to Multi-Cultural Positive Psychological Interventions*, eds L. Van Zyl, and S. Rothmann, Sr. (Cham: Springer), 161–177. doi: 10.1007/978-3-030-20583-6_7^**^.

The authors apologize for this error and state that this does not change the scientific conclusions of the article in any way. The original article has been updated.
